# Retraction

**DOI:** 10.1111/jcmm.15988

**Published:** 2021-01-21

Retraction: Gastrodin relieves inflammation injury induced by lipopolysaccharides in MRC‐5 cells by up‐regulation of miR‐103. Zhuona Xi, Yahong Qiao, Jifang Wang, Jifang Wang, Hongjian Su, Zhen Bao, Hongyan Li, Xiaoming Liao and Xiaolan Zhong. *J Cell Mol Med*. 2020; 24: 1451‐1459.

The above article, published online on 25 November 2019 in Wiley Online Library (https://onlinelibrary.wiley.com/doi/full/10.1111/jcmm.14826), has been retracted by agreement between the journal's Editor in Chief and John Wiley and Sons Ltd. The retraction has been agreed following an investigation based on allegations raised by a third party, and findings that manipulation of backgrounds have occurred for figures 1C, 1F, 2D, 2G, 3A, 3C, 5G and 6D, evidenced by duplications across the backgrounds for different bands. The authors disagree with the decision for retraction.

